# The Editor Burnt out, &c.

**Published:** 1889-04

**Authors:** 


### EDITORIALS AND MISCELLANEOUS.

### THE EDITOR BURNT OUT.

The managing editor of this JOURNAL had his dwelling, his furniture, his library, his instruments, his lecture notes, the MS. of a work nearly ready for the press, and other valuable papers—the labor and accumulation of years—suddenly swept away by fire on the 16th inst. The fact is here mentioned in the hope that all who are in arrears will at once remit their dues. If 8 out of 10 of those who are in arrears will pay up, and advance dues on subscriptions for the present year be forwarded we will be enabled to tide safely over this heavy calamity. We feel that all *true* friends will not only respond to this appeal by remitting their dues, but will use their influence in extending our circulation in their immediate vicinity. "A friend in need is a friend indeed."

